# Primary pulmonary amoebiasis mimicking lung tumour in immunocompromised patient: A case report

**DOI:** 10.1002/rcr2.1199

**Published:** 2023-07-25

**Authors:** Andika Chandra Putra, Bernadina Chyntia, Emon Winardi, Maisie Johan, Albertus Ardian Pradwiyanto, Renaningtyas Tambun, Wiwien Heru Wiyono, Fahmi Alatas

**Affiliations:** ^1^ Department of Pulmonology St. Carolus Hospital Jakarta Indonesia; ^2^ Department of Pulmonology and Respiratory Medicine, Faculty of Medicine Universitas Indonesia Jakarta Indonesia; ^3^ Department of General Medicine St. Carolus Hospital Jakarta Indonesia; ^4^ Department of Internal Medicine St. Carolus Hospital Jakarta Indonesia; ^5^ Department of Radiology St. Carolus Hospital Jakarta Indonesia; ^6^ Department of Anatomy Pathology St. Carolus Hospital Jakarta Indonesia

**Keywords:** Amoebiasis, immunocompromised, parasitic, protozoan, pulmonary amoebiasis

## Abstract

Amoebiasis is the most common protozoan disease caused by *Entamoeba histolytica*. The second most frequent extraintestinal infection, behind amoebic liver abscess, is pulmonary amoebiasis. We present the case of an immunocompromised 40‐year‐old man. He complained of cough for 1 month, shortness of breath, and fever. Chest x‐ray demonstrated left paracardial consolidation, possibly pneumonia or a mass. Chest CT scans with contrast revealed the presence of an abscess‐mimicking tumour. CT‐guided TTB and histology examinations indicated the presence of trophozoites of *E. histolytica*. This patient was diagnosed with pulmonary amoebiasis. Diagnostic criteria for pulmonary amoebiasis include clinical manifestations, radiography, and microscopic examination. There was an improvement in clinical response after a 10‐day course of antibiotics. Amoebiasis of the lungs is treatable with medicines and drainage when necessary. Early diagnosis and treatment are imperative to decrease mortality and morbidity.

## Graphical abstract

This case study explores primary pulmonary amoebiasis, which is a very uncommon condition. The chest imaging showed consolidation that mimicked the tumour, despite the histological analysis identifying the trophozoites or cysts of *Entamoeba histolytica*. Pulmonary amoebiasis is treatable with medicines and drainage if necessary. A multidisciplinary approach and appropriate therapy management are needed to decrease mortality and morbidity.

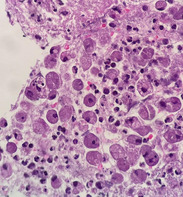

## INTRODUCTION

Amoebiasis is the most common protozoan disease caused by *Entamoeba histolytica*.^1^ It affects about 12% of the world's population, and 50% of cases come from tropical regions such as Mexico, Bangladesh, India, and Indonesia.^2^ The main causative factors are low socioeconomic status, malnutrition, alcoholism, inadequate hygiene, and sanitation. The risk of amoebic liver abscess increases in immunocompromised patients, such as those with HIV infection.^3^ Pleuropulmonary amoebiasis affects 2%–3% of patients, and the inferior and medial lobes of the right lung are the most frequently affected by pulmonary amoebiasis, which typically results from the rupture of an amoebic liver abscess into the pleural space.^2^, ^4^

## CASE REPORT

A 40‐year‐old male presented to a polyclinic with a chief complaint of shortness of breath for 1 month, a productive cough, and a fluctuating fever for the past week. He had been known to have an HIV history since 2004, but he had been lost to follow‐up due to the COVID‐19 pandemic since 2019. His CD4 was 190 during his ARV treatment, after which he stopped regularly taking ARV.

The patient was completely aware (GCS 15). Blood pressure 133/80 mmHg, heart rate 105 bpm, body temperature 37.4°C, respiratory rate 27 bpm, and SpO2 97% with oxygen supplementation of 3 lpm. According to a physical examination, the chest appeared symmetrical with no obvious retraction. By auscultation, there were reduced vesicular breath sounds, particularly in the lower left lung region; bilateral rhonchi at the lung bases; wheezing in both lungs; and by percussion, there was a dullness in the left lung's basal area.

The laboratory findings revealed the following: haemoglobin 9.1 g/dL, erythrocytes 2.94, haematocrit 26.4, leukocytes 11.17 × 10^3^, platelets 277 × 10^3^, basophil 0.2, eosinophil 0.1, neutrophil 69.4%, lymphocytes 17.8, monocytes 12.5, MCV 92, MCH 30, and MCHC 32.6. Respiratory alkalosis was detected by BGA with pH 7.47, pCO2 29.4, pO2 90, and HCO_3_ 21.4.

**FIGURE 1:**
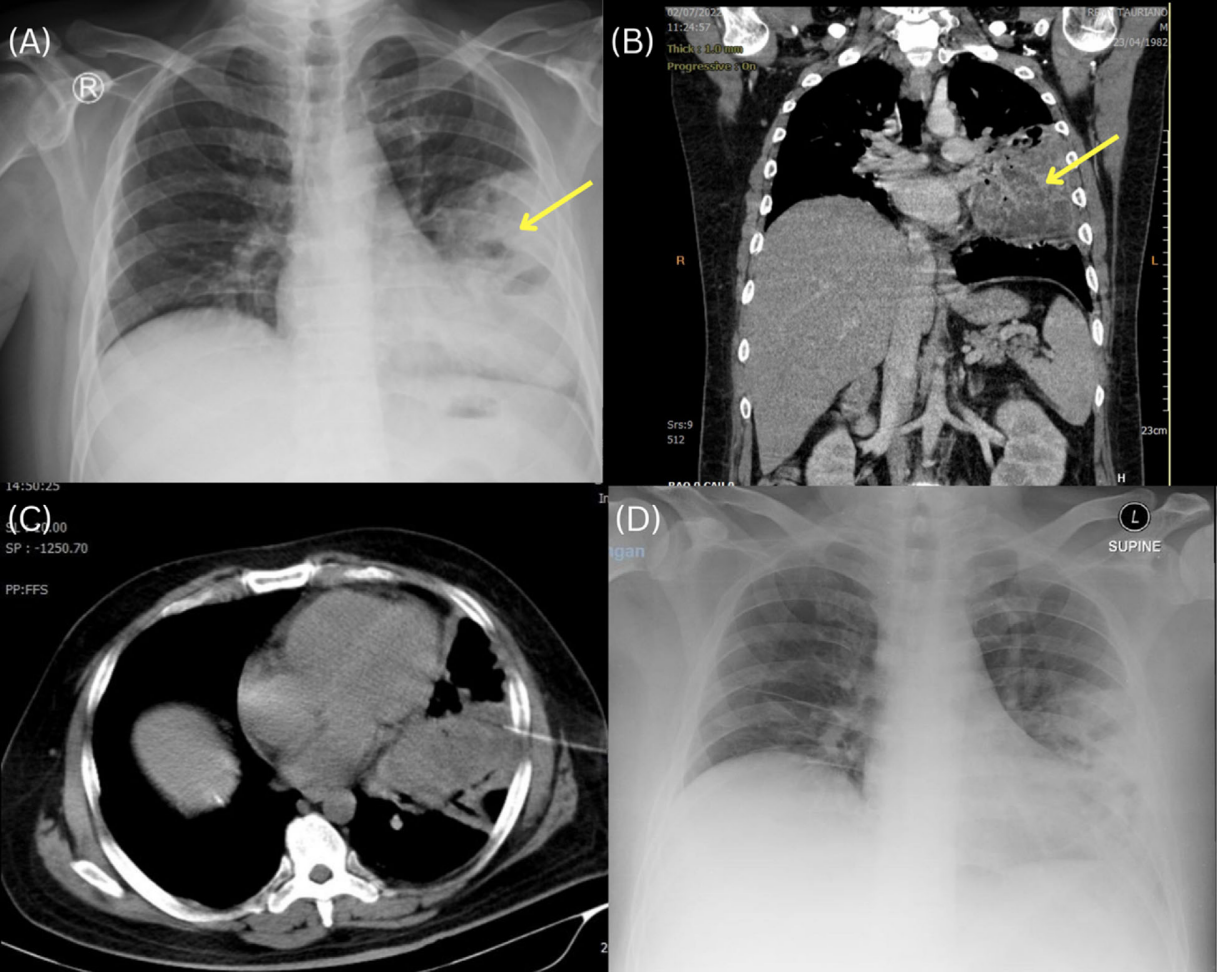
(A) The initial chest x‐ray demonstrates left paracardial consolidation possible pneumonia or mass (arrow). (B) Chest CT scans with contrast, coronal view shows extensive consolidation in the left paracardial with air‐filled areas indicating lung abscess, and suspicious neoplasm (arrow). (C) CT‐guided transthoracic biopsy. (D) Chest x‐ray 1 month after evaluation shows consolidation reduced, relatively thinner.

**FIGURE 2:**
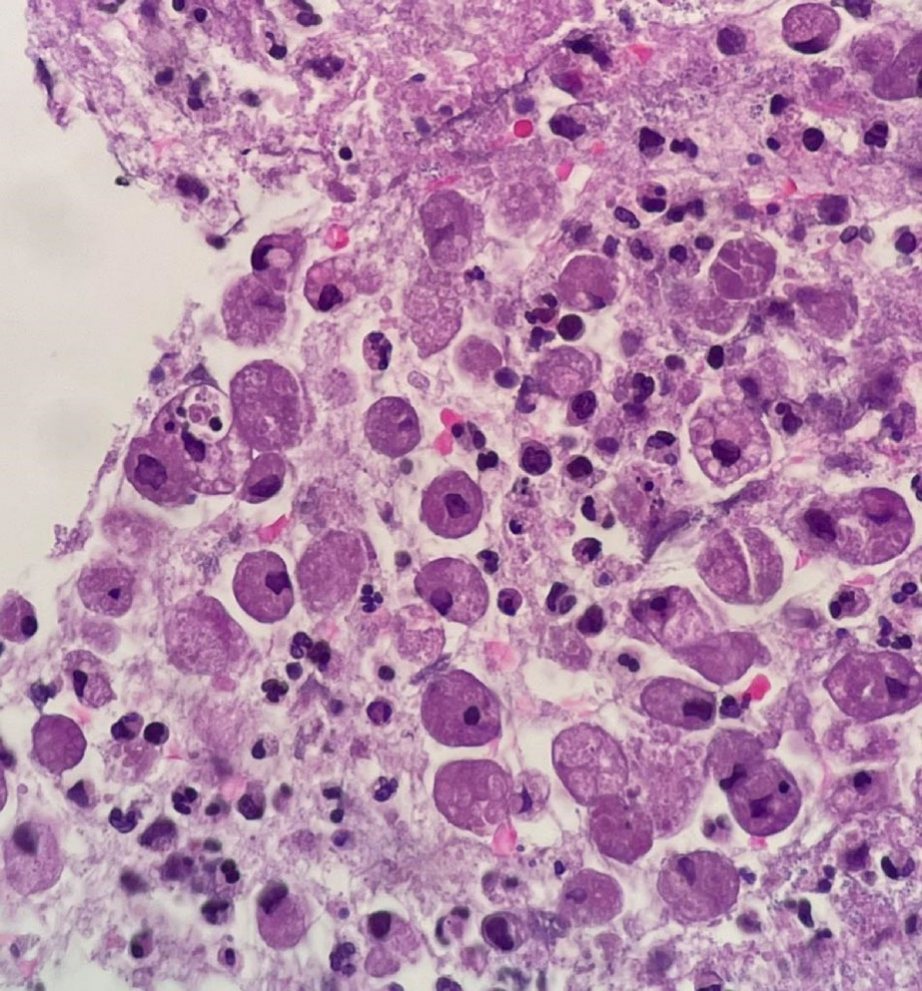
Lung TTB biopsy found colonies of amoebic trophozoites, PMNs, lymphocytes and connective tissue. Histologic suitable to left lung amoebiasis.

Initial chest x‐rays revealed left paracardial consolidation similar to pneumonia or a mass (Figure 1A). The CT scan of the thorax revealed cystic areas with air‐fluid levels, indicating a lung abscess and consolidation of the left lung parenchyma of the superior anterior inferior lobe, indicating a suspicious neoplasm (Figure 1B). Based on the contents of the abscess, a CT‐guided trans‐thoracic biopsy was conducted on the left lateral lung using the Rapid OnSite Examination (ROSE) technique (Figure 1C). The histopathological investigation revealed colonies of Amoeba trophozoites (Figure 2). Histologically, left lung amoebiasis matched.

Following a diagnosis of pulmonary amoebiasis, the patient was administered metronidazole 3 × 500 mg intravenously and ceftazidime 3 × 1 g for 10 days. Twelve days of intravenous corticosteroids were provided. Other symptomatic medications, such as paracetamol, codeine and inhalation, were used by patients to alleviate their fever and cough symptoms. The severity of the symptoms has decreased, which reduced fever, cough and shortness of breath. Following, the vital signs were as follows: blood pressure 115/62 mmHg, pulse 85 bpm, body temperature 36.7°C, respiratory rate 20 bpm, and SpO2 98% without oxygen supplementation. A chest x‐ray 1 month after an evaluation showed consolidation in the parahiler and paracardial left lung reduced, and relatively thinner (Figure 1D).

## DISCUSSION

Several organisms, such as protozoal infections, have been identified as potential opportunistic infections in immunocompromised people.^3^

There are numerous potential mechanisms for pulmonary amoebiasis, first by direct spread of an amoebic liver abscess rupture through the diaphragm. Second, is by intestine infection that spreads to the thoracic cavity either via hematogenous or lymphatic routes.^1^ The inhalation of dust containing *E. histolytica* cysts is another explanation.^4^ Another hypothesis is trophozoites enter the pulmonary circulation via the branches of the middle and inferior hemorrhoidal or vertebral veins into the inferior vena cava to the pulmonary circulation.^5^

Leucocytosis and normocytic normochromic anaemia are frequently found in the laboratory findings of pulmonary amoebiasis.^2^ This is suitable for this patient's laboratory test. Lung x‐ray is the initial line of examination,^4^ thus pulmonary amoebiasis can be suspected when it is discovered lung abscess. The chest x‐ray of this patient was congruent with the findings of the chest CT scan, which showed consolidation, indicating a lung abscess or mass. A CT‐guided TTB with ROSE examination was conducted, and the histopathological investigation revealed amoeba trophozoites.

For the treatment of pulmonary amoebiasis, there are antibiotics, abscess drainage, chest physiotherapy, and surgery for severe infections. The standard treatment consists of intravenous Metronidazole (3 × 750 mg) for 10 days and monitoring of the clinical response and radiological assessment.^1^, ^2^, ^5^ However, drainage is necessary for large pleural effusions (>300 mL) or pericardial effusions. Passive chest physiotherapy helps to eliminate sputum and prevent sputum retention. A thoracotomy or lobectomy is indicated in cases with large abscesses (over 5–10 cm), resistant organisms, haemorrhage, or recurrent disease. In this case, the abscess volume was judged to be minor, and treatment has improved the patient's clinical symptoms and chest x‐ray. Therefore, there was no thoracotomy or lobectomy done.

In conclusion, this case study explores primary pulmonary amoebiasis, which is a very uncommon condition. The chest imaging showed consolidation that mimicked the tumour, despite the histological analysis identifying the trophozoites or cysts of *E. histolytica*. Pulmonary amoebiasis is treatable with medicines and drainage if necessary. A multidisciplinary approach and appropriate therapy management are needed to decrease mortality and morbidity.

## AUTHOR CONTRIBUTIONS

All listed authors contributed to the article.

## CONFLICT OF INTEREST STATEMENT

None declared.

## ETHICS STATEMENT

The author declares that appropriate written informed consent was obtained for the publication of this manuscript and accompanying images.

## Data Availability

The data that support the findings of this study are available on request from the corresponding author. The data are not publicly available due to privacy or ethical restrictions.

## References

[1] Pakrasi R , Mondal A , Kanungo C$A , Chakraborty M . Ruptured amoebic liver abscess into the left lung‐a case report [Internet]. Asian J Case Rep Surg. 2022;5(1):25–31. https://www.sdiarticle5.com/review-history/82048

[2] Dewi K , Suci Y , Dewi I , Iswanto I . Pulmonary amebiasis complicated with massive left empyema and respiratory failure: a case report. Sanamed [Internet]. 2020;15(1):45–49. https://scindeks.ceon.rs/Article.aspx?artid=1452-662X2001045D

[3] Zakaria A , Al‐Share B , Al AK . Primary pulmonary amebiasis complicated with multicystic empyema. Case Rep Pulmonol. 2016;2016:1–4. 10.1155/2016/8709347 PMC4958447 27478673

[4] Aissa A , Hachicha M , Daadoucha A , Ben OI , Aissa S , Barhoumi H , et al. Pleuropulmonary amoebiasis: know to think about. J Pulm Respir Med [Internet]. 2017;7(3):409. https://www.omicsonline.org/open-access/pleuropulmonary-amoebiasis-know-to-think-about-2161-105X-1000409.php?aid=90005

[5] Samuel A , Binoy U , Ali A , Mohan N , Madhu K , Nair R , et al. Primary pulmonary amoebiasis. Pulmon the journal of respiratory sciences [Internet]. 2015;17:122–124. [Accessed October 20, 2022]. https://apccm.in/wp‐content/uploads/2017/02/PULMON‐SEP‐DEC‐15.pdf

